# Research trends on acupuncture for neuropathic pain: A bibliometric analysis from 1979 to 2023

**DOI:** 10.1097/MD.0000000000037962

**Published:** 2024-05-03

**Authors:** Tao Li, Qilu Yan, Wei Huang

**Affiliations:** a Department of Preventive Treatment of Disease, Wenjiang Area Hospital of Traditional Chinese Medicine, Chengdu, China; b Hanyuan Hospital of traditional Chinese Medicine, Yaan, China.

**Keywords:** acupuncture, bibliometric analysis, CiteSpace, neuropathic pain, VOSviewer

## Abstract

**Background:**

Acupuncture has drawn increasing attention as a complementary and alternative therapy for neuropathic pain (NP). The present study aimed to summarize the current status and research trends on acupuncture for NP over the past several decades.

**Methods:**

The publications on acupuncture for NP in the database of Web of Science Core Collection from 1979 to 2023 were searched. VOSviewer (1.6.15) and CiteSpace software (5.5.R2) were applied to identify active authors, journals, countries and institutions, co-cited references and hot keywords.

**Results:**

A total of 642 publications were finally included, and the quantitative trend of annual publications on acupuncture for NP have shown overall upward from 1979 to 2023. Peoples R China was the most productive and influential country, while Kyung Hee University from South Korea was both the first in publications and citations. Fang JQ ranked the first productive author and Han JS was the first 1 among the co-cited authors. The first productive journal was Evidence-based Complementary and Alternative Medicine, while the first co-cited journal was Pain. The high-frequency keywords were divided into 9 clusters, and the frontier topic focused on “Chronic pain”.

**Conclusion:**

This present study visually showed the research status and trends of acupuncture for NP from 1979 to 2023 on the basis of bibliometric analysis, which may in some way help researcher discovery and explore some new research directions and ideas in the future.

## 1. Introduction

Neuropathic pain (NP) occurs frequently after nerve and SCI or disease, causing debilitation of the patient and a decrease in the quality of life.^[1]^ Epidemiological studies showed that the prevalence of NP may be as high as 7% to 8% in the general population.^[2,3]^The mechanism of NP remains unclear. In addition, the analgesics currently available have limited efficacy for this type of pain. Therefore, treatment for NP still remains a big challenge for present medicine around the world, which needs to be solved urgently in the future.^[4]^ According to the World Health Organization, acupuncture has been an officially recognized method of treatment for many kinds of diseases including pain related diseases from 1979.^[5]^ In this context, acupuncture therapy is gradually becoming popular and increasingly accepted in a lot of countries all around the world.^[6]^ Data have shown that acupuncture can relief NP without causing dependency.^[7]^ A prospective exploratory pilot study shows that EA can decrease the intensity of NP, in particular, such as burning, electric shock-like pain, and mechanical hyperalgesia.^[8]^ Another exploratory study indicated that acupuncture treatment can relieve NP and few side effects were reported.^[9]^ As a necessary complementary and alternative therapy, especially in the field of analgesia, acupuncture has attracted increasing international attention.

Despite of widely and increasingly used as complementary and alternative therapy for NP, acupuncture remains grossly underutilized and its mechanism is also still completely clear. Therefore, a specific and accurate overview of the research status, development trends and frontiers is meaningful for understanding the influence of acupuncture for NP. With the characteristics of quantitatively measuring the productivity, inter-relationships and development trends in a certain field, bibliometric analysis is now becoming popular in a variety fields. Using bibliometric tools, analysis of co-authorship, co-citation, and co-occurrence can visually displayed established and emerging research areas.

In this study, we applied a bibliometric analysis by using CiteSpace (5.5.R2) and VOSviewer (1.6.15) to reveal the research status, development trends and frontiers in the field of acupuncture for NP from 1979 to 2023.

## 2. Methods

### 2.1. Data acquisition and search query strategy

**Figure 1.**
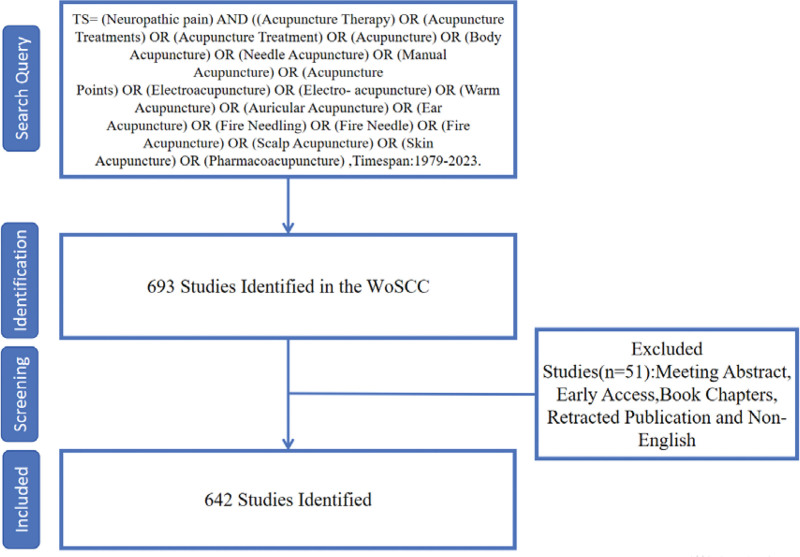
Flowchart of literature selection.

Research data were retrieved and download from Web of Science Core Collection, including Science Citation Index Expanded, Social Sciences Citation Index, Conference Proceedings Citation Index-Science, Current Chemical Reactions, and Index Chemicus on January 17, 2024. All data only concerned on the literature published from 1979 to 2023 with specific Strategies shown in table 1. The data acquisition was implemented independently by 2 reviewers (TL and WH), and any discrepancy was resolved by consultation with a third reviewer. Only English articles and reviews were included. Preliminarily, there were a total of 693 articles retrieved, however, 51 irrelevant articles including meeting abstracts, early access, book chapters, retracted publication and non-English studies were excluded (Fig. 1). Finally, 642 publications in total were identified and the retrieved ones were exported in the form of all records and references, saved as plain text files in the format as “download_XX.txt”.

**Table 1:** The Topic search query.

Set	Search Query	Results	Timespan	Data
#1	TS = Neuropathic pain	43,794	1979–2023	SCI-EXPANDED, SSCI, CPCI-S, CCR-EXPANDED and IC.
#2	TS = (Acupuncture Therapy) OR (Acupuncture Treatments) OR (Acupuncture Treatment) OR (Acupuncture) OR (Body Acupuncture) OR (Needle Acupuncture) OR (Manual Acupuncture) OR (Acupuncture Points) OR (Electroacupuncture) OR (Electroacupuncture) OR (Warm Acupuncture) OR (Auricular Acupuncture) OR (Ear Acupuncture) OR (Fire Needling) OR (Fire Needle) OR (Fire Acupuncture) OR (Scalp Acupuncture) OR (Skin Acupuncture) OR (Pharmacoacupuncture)	29,789		
#3	#1AND#2	693		

CCR-EXPANDED = current chemical reactions, CPCI-S = conference proceedings citation index-science, IC = index chemicus, SCI-EXPANDED = science citation index expanded, SSCI = social sciences citation index, TS = topic.

### 2.2. Data analysis

VOSviewer (1.6.15) and CiteSpace (5.5.R2) software were applied to analyze all included data. VOSviewer^[10]^ was used to construct co-occurrence maps and identify active authors, countries and regions, institutions, and journals, as well as the top co-cited journals, authors and references. CiteSpace^[11]^ was applied to identify the keywords clustering and burst keywords. The parameters of VOSviewer was set according to default mode: Choose type of data: Create a map based on bibliographic data (choose this option to create a co-authorship, keywords co-occurrence, citation, bibliographic coupling, or co-citation map based on bibliographic data); Choose data source: Read data from bibliographic database files; Select files: Web of Science; Choose type of analysis and counting method: Co-authorship: including “Unit of analysis authors, organizations and countries”; counting method: full counting. Co-occurrence: Choose unit of analysis all keywords and choose full counting in the counting method. Citation: choose “Unit of analysis sources. Co-citation: including ‘Unit of analysis cited references, cited sources and cite authors.’; counting method: full counting. CiteSpace was setting as the followings: Time Span: 1979 to 2023, ‘1’ was selecting as Years Per Slice; Term source: defaulting all types, including ‘Tittle’, ”Abstract,” ”Author keywords,” and ” Keywords Plus”; Node type: a single node type in sequence were author, country, institution, and keywords; Links: choosing “Cosine” as strength and “Within Slices” as Scope; Selection criteria: The most cited or occurred items from each slice were selected top 50 levels; Pruning: choosing ”pathfinder.” Moreover, on the basis of the 2023 Journal Citation Reports released by Clarivate Analytics, journal impact factors (IF) were identified.

## 3. Results

### 3.1. Annual publications

**Figure 2.**
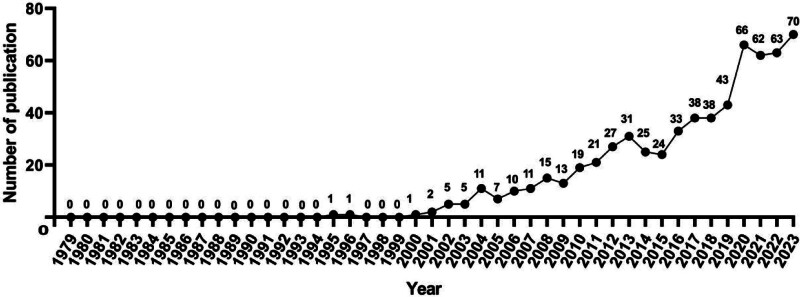
The annual number of publications on acupuncture for NP from 1979 to 2023. NP = neuropathic pain.

According to statistics, a total of 642 articles were finally included on the basis of the retrieval methods. The specific number of annual publications on acupuncture for NP was shown in Figure 2. It was obvious that development of acupuncture for NP can be divided into 2 stages. From 1979 to 1999, there were only 2 publications in the field, however, annual publications had been generally increasing from 2000 to 2023, indicating a steady development of acupuncture for NP after 2000. In addition, the number of publication reached the its peak (n = 70) in 2023.

### 3.2. Analysis of authors and co-cited authors

**Figure 3.**
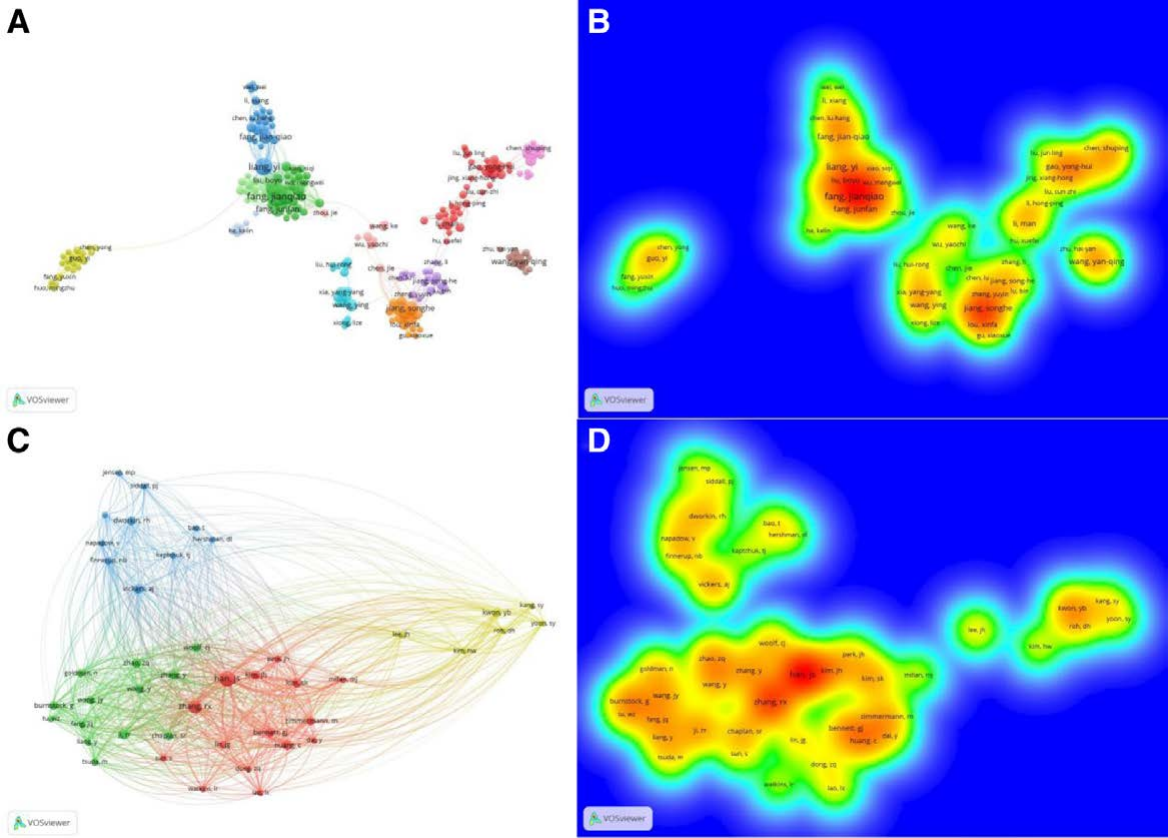
The visualization of authors and co-cited authors. (A) VOSviewer visualization map of co-authorship related to research on acupuncture for NP. (B) The density map of authors. The closer the keyword node color is to red, the higher the frequency of its co-occurrence. (C) VOSviewer visualization map of co-cited authors related to research on acupuncture for NP. (D) The density map of co-cited authors. The closer the keyword node color is to red, the higher the frequency of its co-citation.

A total of 3103 authors were involved in the research field of acupuncture for NP, among which 495 authors published more than 2 articles (T ≥ 2). Fang JQ ranked first in high yield authors (n = 19), then followed by Kim SK (n = 17), Liang Y (n = 15) and Shao XM (n = 13). The remaining 6 authors were published 10 to 11 publications respectively shown in Table 2. The authors (n = 492) who published more than 2 articles (T ≥ 2) were selected, and then the network map of authors were drew out. As shown in Figure 3A, the active authors formed 12 clusters, in which the node represented author and the link showed the cooperation between each one. Moreover, same color node assembled into a cluster. These clusters represented active collaborations in the research field of acupuncture for NP. Figure 3B showed the density map of authors, and this map could visually and clearly display the hot authors. It is obviously shown in Figure 3B that Fang JQ had the hottest color which represented his most active in the field.

**Table 2:** Top 10 authors and co-cited authors.

Author	Documents	Co-cited author	Citations
Fang JQ	19	Han JS	239
Kim SK	17	Zhang RX	178
Liang Y	15	Ji RR	97
Shao XM	13	Huang C	95
Du JY	11	Zhao ZQ	94
Min BI	11	Vickers AJ	94
Lin YW	11	Kwon YB	91
Jiang SH	10	Kim SK	90
Tu WZ	10	Zhang Y	85
Roh DH	10	Burnstock G	84

Co-cited authors are authors co-cited in a series of publications. According to statistics, there were 44 authors co-cited over 40 among 16,713 co-cited authors from all 642 articles. Han JS (n = 239) ranked first, followed by Zhang RX (n = 178), Ji RR (n = 97), Huang C (n = 95), Zhao ZQ (n = 94) and Vickers AJ (n = 94). The following 4 co-cited authors with co-citations ranging from 84 to 91 listed in Table 2. Figure 3C and Figure 3D showed co-cited authors and the density map of co-cited authors (n = 44 with high co-citations (T ≥ 40), and this map could visually and clearly display the hot co-cited authors. It is obvious that Han JS had the hottest color which represented for the most co-cited.

### 3.3. Distribution of countries and regions

**Figure 4.**
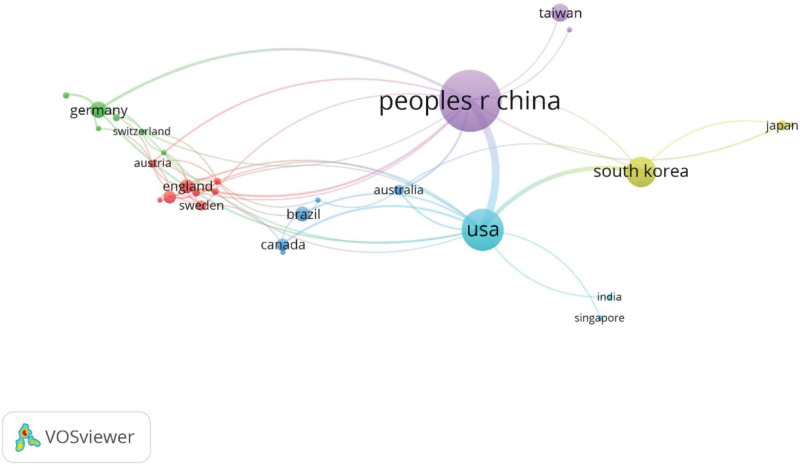
The co-authorship network visualization map of countries and regions on acupuncture for NP. NP = neuropathic pain.

Statistically analysis showed that all 642 publications were coauthored from 44 countries and regions, among which 29 countries and regions published over 2 articles, shown in Figure 4. In addition, Table 3 presented the top 10 productive countries and regions, in which Peoples R China was the first prolific country with 326 publications (n = 326), then followed by USA (n = 146), South Korea (n = 80), Chinese Tai Wan (n = 28) and Germany (n = 24). Obviously, China ranks first both in publications and citations in the research of acupuncture for NP.

**Table 3:** Top 10 publications and citations of countries/regions related to acupuncture for NP.

Rank	Country/Region	Publications	Citations	Proportion (%)
1	Peoples R China	326	5379	50.77
2	USA	146	4852	22.74
3	South Korea	80	1964	12.46
4	Chinese Tai Wan	28	688	4.36
5	Germany	24	417	3.73
6	Brazil	19	279	2.95
7	Italy	17	1440	2.64
8	Sweden	14	749	2.18
9	Canada	14	460	2.18
10	Australia	9	108	1.40

NP = neuropathic pain.

### 3.4. Distribution of institutions

**Figure 5.**
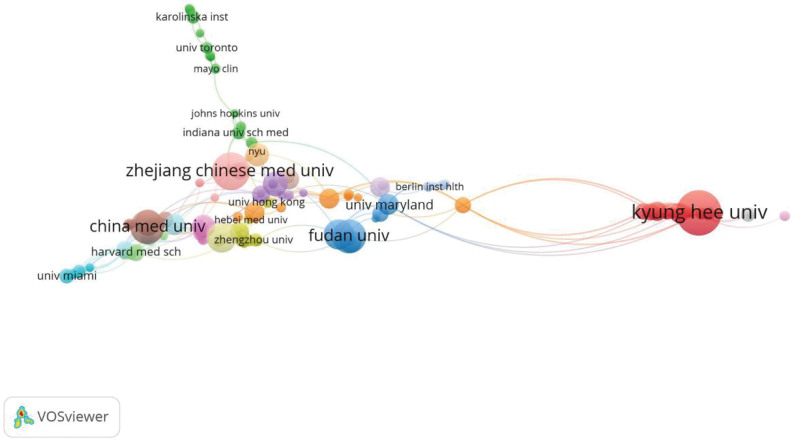
The co-authorship network visualization map of institutions on acupuncture for NP. NP = neuropathic pain.

According to VOSviewer analysis, 642 articles were published by 745 different institutions, and there were 179 institutions publishing at least 2 documents (T ≥ 2). The co-occurrence of institutions was displayed in Figure 5, and Table 4 listed the top 10 active institutions. Kyung Hee University was the first prolific institution (n = 56), followed by Zhejiang Chinese Medical University (n = 38), China Medical University and Fudan University (n = 31), Chinese Academy of Medical Sciences (n = 24) and Shanghai University of Traditional Chinese Medicine (n = 24). Among the top 10 organizations, There are 9 Chinese institutions. However, Kyung Hee University was ranking first both in publications and citations.

**Table 4:** Top 10 publications and citations of institutions related to acupuncture for NP.

Rank	Institution	Publications	Citations	Country
1	Kyung Hee University	56	1499	South Korea
2	Zhejiang Chinese Medical University	38	661	China
3	China Medical University	31	594	China
4	Fudan University	31	717	China
5	Chinese Academy of Medical Sciences	24	379	China
6	Shanghai University of Traditional Chinese Medicine	24	303	China
7	China Medical University Hospital	19	313	China
8	Chengdu University of Traditional Chinese Medicine	17	316	China
9	Peking University	15	778	China
10	Wenzhou Medical University	15	184	China

NP = neuropathic pain.

### 3.5. Analysis of journals and co-cited journals

All included articles were published in 260 journals, and there were 32 ones published more than 5 articles. In addition, there were a total of 4298 co-cited journals. The impact factor (IF) and journal quartile were referred to Journal Citation Reports 2023 (JCR 2023). Top 10 prolific and co-cited journals were respectively showed in Table 5. Obviously, the top 3 prolific journals were Evidence-based Complementary and Alternative Medicine (n = 40, NO IF), Acupuncture in Medicine (n = 27, IF = 2.5), and Frontiers in Neuroscience (n = 23, IF = 4.3), while the top 3 co-cited ones were Pain (IF = 7.4), Brain Research (IF = 2.9), and Evidence-based Complementary and Alternative Medicine (NO IF).

**Table 5:** Top 10 scholarly journals and co-cited journals related to acupuncture for NP.

Rank	Journal	Publications	IF (Quartile)	Co-cited journal	Citations	IF (Quartile)
1	Evidence-based Complementary and Alternative Medicine	40	–	Pain	2406	7.4 (Q1)
2	Acupuncture in Medicine	27	2.5 (Q3)	Brain Research	809	2.9 (Q3)
3	Frontiers in Neuroscience	23	4.3 (Q2)	Evidence-based Complementary and Alternative Medicine	690	-
4	Journal of Pain Research	18	2.7 (Q3)	Journal of Neuroscience	685	5.3 (Q1)
5	Journal of Pain	16	4.0 (Q2)	Journal of Pain	639	4.0 (Q2)
6	Medicine	14	1.6 (Q3)	Neuroscience Letters	634	2.5 (Q3)
7	Toxins	11	4.2 (Q2)	Neuroscience	401	3.3 (Q3)
8	Brain Research Bulletin	10	3.8 (Q2)	Acupuncture in Medicine	384	2.5 (Q3)
9	Neuroscience Letters	9	2.5 (Q3)	Molecular Pain	379	3.3 (Q3)
10	Brain Research	9	2.9 (Q3)	Brain Research Bulletin	377	3.8 (Q2)

IF = impact factor, NP = neuropathic pain.

### 3.6. Analysis of co-cited references

Co-cited references are references co-cited in a series of publications. Ultimately, a total of 23,915 references were co-cited by all 642 publications, and only 52 references with co-citation frequency over 20 times met the thresholds (T ≥ 20).Table 6 showed out top 10 co-cited references. With 94 co-citations, the study from Zhang ZQ ranked first, and this study expounded neural mechanism underlying acupuncture analgesia.^[12]^ The study from Han JS published in Trends in Neurosciences ranked second, which indicated that electrical stimulation of different frequencies can lead to release different neuropeptides.^[13]^ Zhang RX’s study ranked third among top 10 co-cited references, which showed the mechanisms of acupuncture-electroacupuncture on persistent pain.^[14]^ The following 7 references had co-citations ranging from 39 to 67.^[15–21]^ In general, the contents of these top10 high co-cited references mainly focus on the mechanism of acupuncture analgesia.

**Table 6:** Top 10 co-cited reference with high co-citations related to acupuncture for NP.

Rank	Co-cited reference	Journal	Published year	Co-citations
1	Neural mechanism underlying acupuncture analgesia.	Prog Neurobiol	2008	94
2	Acupuncture: neuropeptide release produced by electrical stimulation of different frequencies.	Trends In Neurosciences	2003	92
3	Mechanisms of acupuncture-electroacupuncture on persistent pain.	Anesthesiology	2014	72
4	Quantitative assessment of tactile allodynia in the rat paw.	J Neurosci Methods	1994	67
5	A peripheral mononeuropathy in rat that produces disorders of pain sensation like those seen in man.	Pain	1988	67
6	Adenosine A1 receptors mediate local antinociceptive effects of acupuncture.	NAT Neurosci	2010	62
7	Ethical guidelines for investigations of experimental pain in conscious animals	Pain	1983	57
8	Effects of electroacupuncture on cold allodynia in a rat model of neuropathic pain: mediation by spinal adrenergic and serotonergic receptors.	Exp Neurol	2005	56
9	Relieving effects of electroacupuncture on mechanical allodynia in neuropathic pain model of inferior caudal trunk injury in rat: mediation by spinal opioid receptors	Brain Research	2004	47
10	The effect of electroacupuncture on pain behaviors and noxious stimulus-evoked Fos expression in a rat model of neuropathic pain	Journal of Pain	2001	39

NP = neuropathic pain.

### 3.7. Analysis of keywords

**Figure 6.**
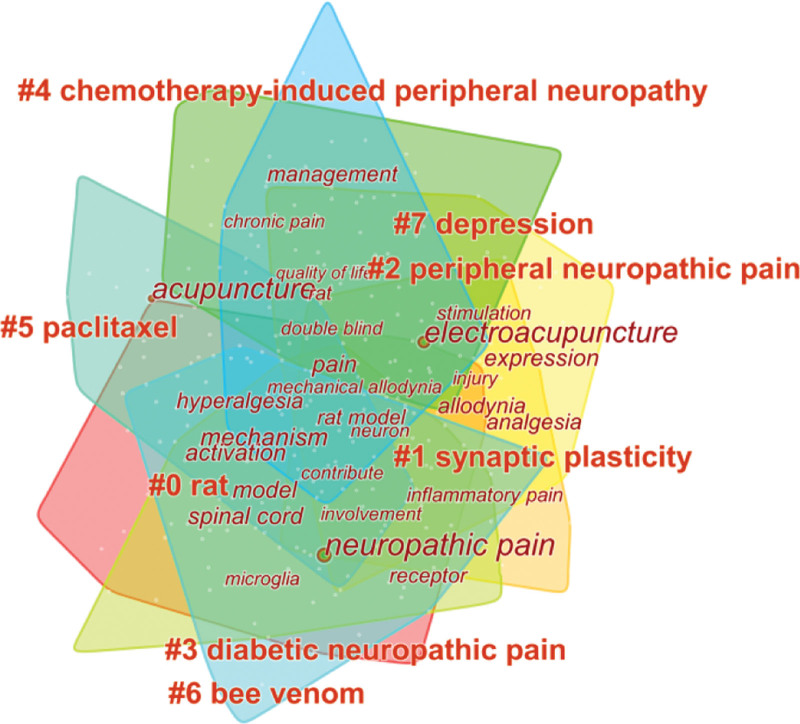
Map of keywords co-occurrence and cluster related to acupuncture for NP from 1979 to 2023. NP = neuropathic pain.

**Figure 7.**
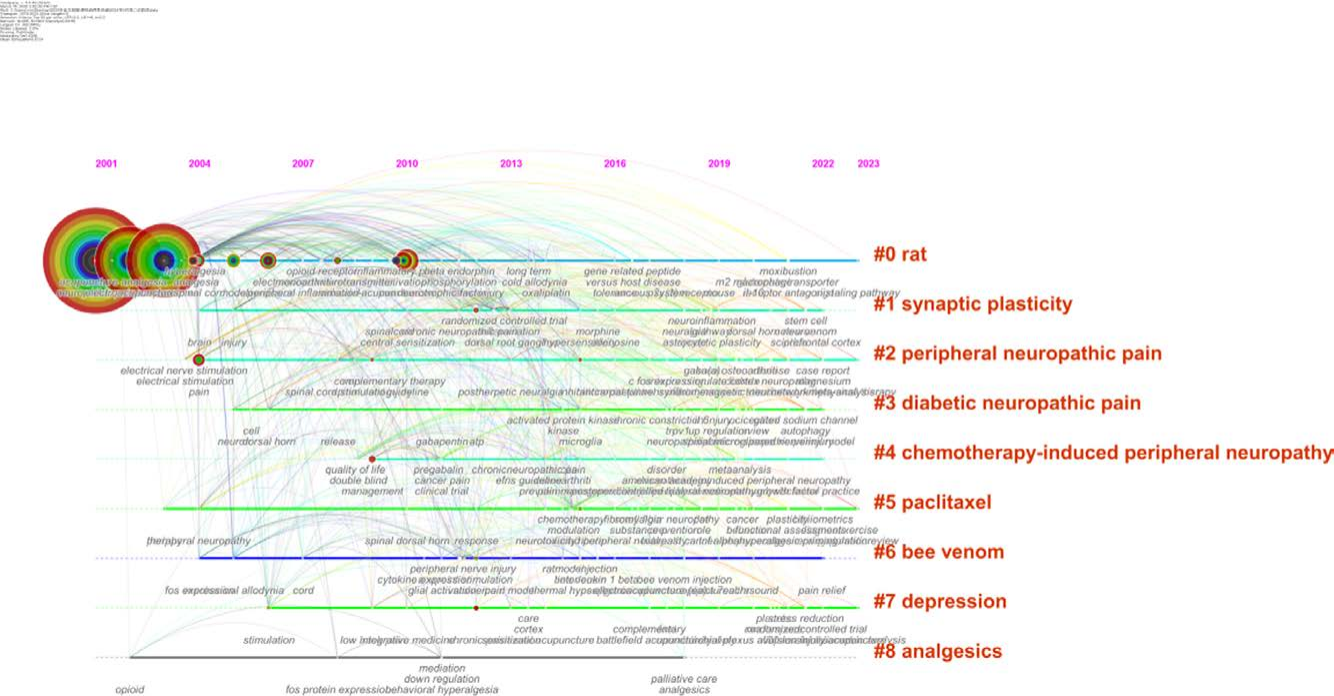
Map of the timeline view of each cluster related to acupuncture for NP from 1979 to 2023. NP = neuropathic pain.

Statistically analysis showed that a total of 2740 keywords came from all 642 publications, Table 7 showed the top 10 keywords with high frequency and centrality related to acupuncture for NP. In terms of frequency, “NP” ranked first in frequency at 373, followed by “Acupuncture,” “Electroacupuncture,” “Mechanism” and “Pain.” In addition, the top 5 keywords with high centrality were “Allodynia” (0.15), “Pain” (0.11), “Spinal cord” (0.10), “Prevalence” (0.10) and “Rat” (0.09). As the key information of publications, numerous keywords can identify the hotspots and trends of a certain field. After a comprehensive analysis of keywords by CiteSpace, 9 clusters were identified shown in Figure 6, and the cluster labels were #0 rat, #1 synaptic plasticity, #2 peripheral neuropathic pain protocol, #3 DNP, #4 chemotherapy-induced peripheral neuropathy (CIPN), #5 paclitaxel neuroinflammation, #6 bee venom, #7 depression #8 analgesics. Meanwhile, this map of keywords co-occurrence and cluster indicated that the modularity score was 0.4208 and the mean silhouette score was 0.5731. It is credible and reliable when the modularity score and mean silhouette score over 0.3 and 0.5 respectively.^[22]^ Therefore, the clustering result in this study was credible and reliable. In addition, Figure 7 showed the timeline view of each cluster, in which “#0 rat” appeared earliest and it has continued to nowadays, in addition, “#4 CIPN” appeared the latest during keywords clusters. Time provided the insights into the temporal trends and dynamics within the field of interest.

**Table 7:** Top 10 Frequency and Centrality of Keywords Related to Acupuncture for NP.

Frequency	Keywords	Centrality	Keywords
373	Neuropathic Pain	0.15	Allodynia
275	Acupuncture	0.11	Pain
254	Electroacupuncture	0.10	Spinal cord
105	Mechanism	0.10	Prevalence
85	Pain	0.09	Rat
83	Activation	0.09	Double blind
81	Spinal cord	0.08	Management
80	Expression	0.08	Modulation
79	Model	0.07	Analgesia
66	Hyperalgesia	0.07	Mechanical allodynia

NP = neuropathic pain.

**Figure 8.**
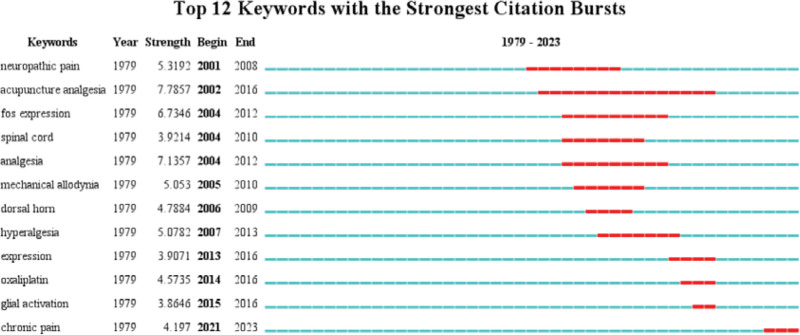
Top 12 Keywords with the strongest citation bursts.

Burst keywords are derived from a great quantity of keywords. Among a total of 2740 keywords, 12 burst keywords with high strength citation were identified, which were displayed in Figure 8. Development of research frontiers about acupuncture for NP between 1979 and 2023 can be revealed by the evolution of keywords in these publications. The strongest citation burst word with strength of 7.7857 is “acupuncture analgesia,” which appeared in 2002 and continued to 2016. In addition, keywords bursting mainly concentrated on the period of 2002 to 2016. “Chonic pain” were the most recent 1 with the strength of citation burst of 4.197 from 2021 to 2023.

## 4. Discussion

### 4.1. General information

By using bibliometric methods, this present study analyzed publications related to acupuncture for NP. The overall rising curve of the number of annual publications indicated that researches on acupuncture for NP are attracting increasing attention, especially after 2000, even with some fluctuations. Analysis of geographical distribution provide an obvious regional characteristic that Peoples R China ranks first in publications and citations in the research of acupuncture for NP, which suggested that Peoples R China was the core country in the research field. In addition, Top 10 institutions only came from Peoples R China and South Korea, in which Kyung Hee University was both the first in publications and citations. Moreover, among 3103 authors and 16,713 co-cited authors, Fang JQ and Han JS were the most productive and co-cited author respectively. High productive and co-cited authors have an important influence and positively promote the development of the research field on acupuncture for NP. On the basis of analyzing journals and co-cited journals, we found that the first productive journal was Evidence-based Complementary and Alternative Medicine, while the first co-cited journal was Pain. However, IF of top 10 productive and co-cited journals were mainly located in Q2 or Q3, suggesting that improving research quality while increasing output may contribute to enhancing their academic influence.^[23]^ References played a crucial role in the selection, execution, and summary of scientific research,^[24]^ furthermore, high co-cited references had an important status and influence in some field. Co-cited references on mechanism research had crucial academic influence in the field of acupuncture for NP.

### 4.2. Research hotspots and trends

High-frequency keywords may in a sense reflect research hot spots.^[25]^ According to the top 10 frequency and centrality keywords in Table 7 and clustering analysis of high-frequency keywords in Figure 6, research hotspots on acupuncture for NP were mainly summarized in the following 2 aspects:

#### 4.2.1. Mechanism of acupuncture analgesia

Although acupuncture is widely used in the treatment of pain, its analgesic mechanism remains largely unknown. So far, a plenty of studies have implicated neural mechanism is the main mechanism of acupuncture analgesia. It is found that acupuncture signals mainly ascend to brain via neural pathways mediated by diverse neurotransmitter or signal molecules, such as opioid peptides, glutamate, 5-hydroxytryptamine, and cholecystokinin octapeptide. And then the signals caused positive changes in different brain regions, which was found that a network in the brain regulated and integrated pain, including the nucleus raphe magnus, cingulate cortex, amygdala, thalamus, periaqueductal gray, caudate nucleus, arcuate nucleus, posterior parietal cortex, preoptic area, nucleus submedius, habenular nucleus, insula, prefrontal cortex, locus coeruleus, accumbens nucleus, septal area, and other brain areas.^[12,26,27]^ In addition, by using functional magnetic resonance imaging, these changes in different brain regions is well observed and proved after acupuncture. A systematic review showed that the preventive effect of acupuncture on migraine may be achieved by regulating the visual network, default mode network, sensory motor network, frontoparietal network, limbic system, and/or descending pain modulatory system.^[28]^ Moreover, in the research field of cytology, recent studies showed some new discoveries on mechanism of acupuncture analgesia. The activation of mast cells under acupuncture stimuli have been reported.^[29–31]^ Acupuncture stimulation can activate the transient receptor potential vanilloid (TRPV) channels (including TRPV1 and TRPV2) on mast cells, and then bioactive substances (such as their degranulation) were released, so as to activate nerve receptors to produce analgesic effect.^[32]^

#### 4.2.2. Acupuncture for many types of neuropathic pain

Including many types, NP mainly divided into central neuropathic pain and peripheral neuropathic pain. As a severe disease of the central nervous system, spinal cord injury (SCI) often caused central neuropathic pain.^[33]^ However, an accumulating body of research found that acupuncture can effectively improve NP related to SCI. On the 1 hand, acupuncture may inhibiting apoptosis caused by SCI, including oligodendrocytes and microglia.^[34]^ On the other hand, acupuncture may promote nerve repair and regeneration to reduce NP caused by SCI. A 2021 study found that EA promoted axonal regeneration by inhibiting the Rho/ROCK signaling pathway.^[35]^

Acupuncture also has good effects for peripheral neuropathic pain, especially for diabetic neuropathic pain (DNP) and postoperative pain. As a common complication of diabetes mellitus, diabetic peripheral neuropathy can lead to DNP. A randomized study found that acupuncture may lead to a reduction in neurological deficits and thus alleviate DNP.^[36]^ Besides precipitated by peripheral neuropathy, NP is also common after operation. Although perioperative analgesic methods are used, injury to nerve is still frequent among kinds of surgeries and can result in chronic pain.^[37]^ Recent research found that acupuncture cannot provide a safe analgesic supplement or alternative, but also reduce the use of analgesics and their side effects.^[38,39]^ Furthermore, it is noteworthy that chemotherapeutic drugs, such as paclitaxel, often induce CIPN, including allodynia, especially NP. However, drugs currently used to alleviate the allodynia often cause many side effects, whose application is restricted.^[40]^ A growing body of research indicates that acupuncture used for CIPN is safe and effective.^[41–43]^ Not only can acupuncture alleviate the NP of CIPN and improve touch perception thresholds,^[44]^ but also improve the outcome measures, including quality of life, Neuropathic Pain Scale, Nerve Conduction Velocity, Visual Analogue Scale, Numerical Rating Scale, Functional Assessment of Cancer Therapy/Gynecologic Oncology Group-Neurotoxicity, National Cancer Institute-Common Toxicity Criteria for Adverse Events.^[45–47]^

### 4.3. Research frontiers

Burst keywords were the keywords burst out at a particular time, which may reflect the evolution of hot spots and forecast the research trend of a certain field. In this present study, burst keywords were identified by application of CiteSpace. According to the analysis of burst keywords, “chronic pain” was identified as current frontier of related research. Compared to other types of chronic pain, NP is more complex, has longer course, and can lead to greater physical and mental damage.^[48]^ Therefore, treatment of NP is still also a long-lasting process, and need to be managed, including early treatment of NP, optimal dosage of analgesic drugs, addition of opioids, and application of acupuncture.^[49]^

## 5. Limitations

Due to the limitations of software, the present study only included the articles in WoSCC and the language were limited in English, whereas those in other language and other databases, such as Chinese database, Scopus and PubMed, had not been included. Therefore, only 642 articles included do not completely contain all information related to the research field, which is the main limitation of the present study. Despite the limitation, we are still confident that the present study has given an integrated overview of the research status and trends in the field of acupuncture for NP, for WoSCC is one of the biggest databases, in which there is a great quantity of high-quality publications in worldwide.

## 6. Conclusion

The curve on number of annual publications suggested that more attention has been paid to acupuncture for NP. In addition, with the help of bibliometric analysis, the top active authors, institutions, journals, countries and regions were determined. Analysing of co-cited references shed light on top articles with important influence in the research field. High-frequency keywords can reveal the research hotspots of the field, and the burst keywords may show out the development trends and frontiers. To conclude, the present study successfully reveals the research status and hot spots from 1979 to 2023, as well as research trends and frontiers on acupuncture for NP. Moreover, in some way, it may give some reference for future research.

## Acknowledgments

The authors would like to express their appreciation to Professor Chen CM, Van Eck and Waltman L, who invented CiteSpace and VOSviewer.

## Author contributions

**Conceptualization:** Tao Li.

**Data curation:** Tao Li.

**Formal analysis:** Tao Li.

**Methodology:** Tao Li.

**Resources:** Tao Li.

**Software:** Tao Li.

**Writing – original draft:** Tao Li, Qilu Yan.

**Writing – review & editing:** Tao Li, Qilu Yan, Wei Huang.
